# Association of current HbA1c with concurrent multiorgan screening abnormalities and the incremental value of routine clinical characteristics in type 2 diabetes: a single-center cross-sectional study in Wenzhou, China

**DOI:** 10.3389/fendo.2026.1974690

**Published:** 2026-10-07

**Authors:** Chengcheng Ye, Huahua Peng, Xinxin Wang, Yongsheng Wang

**Affiliations:** Wenzhou Hospital of Integrated Traditional Chinese and Western Medicine, Wenzhou, China

**Keywords:** albuminuria, arterial stiffness, HbA1c, multiorgan screening abnormalities, peripheral neuropathy screening, type 2 diabetes mellitus

## Abstract

**Background:**

Glycated hemoglobin A1c (HbA1c) is central to glycemic management but mainly reflects recent average glycemia rather than long-term cumulative exposure or existing organ abnormalities. We assessed whether adding age, sex, diabetes duration, body mass index (BMI), and systolic blood pressure to HbA1c improved the identification of concurrent vascular, renal, and peripheral nerve abnormalities in patients with type 2 diabetes mellitus (T2DM).

**Methods:**

This was a single-center cross-sectional analysis of a prospectively maintained clinical registry at a standardized metabolic disease management center. Adults with T2DM assessed between January 10, 2022, and April 30, 2026, were included, and each patient contributed one standardized visit selected for analysis (the index assessment). The primary outcome was a screening abnormality in at least two of three organ domains: vascular, renal, and peripheral nerve. We used multivariable logistic regression to estimate adjusted cross-sectional associations and 20 repeats of stratified fivefold cross-validation to assess the discrimination and calibration of three prespecified nested models.

**Results:**

The analysis included 674 patients with a mean age of 52.6 years, of whom 62.9% were men. Among them, 342 (50.7%) had abnormalities in at least two organ domains. Of the 112 patients with current HbA1c <7.0%, 44 (39.3%) had multiorgan abnormalities. Current HbA1c was not clearly associated with the outcome after adjustment (adjusted odds ratio [OR] per 1-percentage-point increase, 1.04; 95% confidence interval [CI], 0.96–1.13), whereas age, diabetes duration, and systolic blood pressure were independently associated with it. The cross-validated area under the receiver operating characteristic curve (AUC) was 0.523 (95% CI, 0.479–0.568) for HbA1c alone and 0.775 (95% CI, 0.740–0.810) after age, sex, diabetes duration, BMI, and systolic blood pressure were added. The difference in AUC was 0.251 (95% CI, 0.195–0.304).

**Conclusions:**

A single current HbA1c measurement had limited discriminatory ability for concurrent clustering of multiorgan screening abnormalities in adults with T2DM. Adding age, sex, diabetes duration, BMI, and systolic blood pressure to HbA1c substantially improved discrimination of this clustering phenotype. Age, diabetes duration, and systolic blood pressure were independently associated with multiorgan abnormalities.

## 1 Introduction

Type 2 diabetes mellitus (T2DM) is one of the most common chronic metabolic diseases worldwide. In 2024, the global prevalence of diabetes among adults aged 20–79 years was approximately 11.1%, corresponding to an estimated 589 million individuals (1). Its long-term disease burden arises not only from persistent metabolic abnormalities but also from involvement of multiple organ systems, including the vascular, renal, and peripheral nervous systems. Contemporary management of T2DM extends beyond glycemic control and includes regular assessment of cardiovascular risk, screening for kidney disease and peripheral neuropathy, and multifactorial intervention. Current clinical practice recommends periodic assessment of urinary albumin-to-creatinine ratio and estimated glomerular filtration rate, peripheral nerve function and foot risk, and cardiovascular risk factors (2–5). Management may include individualized glucose-lowering therapy, lifestyle intervention, blood pressure and lipid control, and kidney- and cardiovascular-protective medications when clinically indicated. These interventions may influence measurements obtained during subsequent clinical assessments without fully reversing pre-existing organ injury. Because abnormalities across organ systems share risk factors such as age, diabetes duration, hyperglycemia, and blood pressure, they may therefore cluster within the same individual (6–8). However, reporting abnormality rates separately for individual organ systems does not capture their coexistence within a patient and may underestimate the overall burden of concurrent multiorgan involvement.

Glycated hemoglobin A1c (HbA1c) is a core measure for assessing recent mean glycemia and setting individualized treatment targets and primarily reflects glycemic exposure during the preceding 2–3 months (9). Previous studies have established that early and sustained glycemic control confers long-term benefits in preventing complications (10, 11). Nevertheless, existing research has focused mainly on associations of HbA1c or long-term glycemic trajectories with single-organ outcomes or on pairwise relationships between complications (6–8). The overall burden, co-occurrence patterns, and identification of concurrent screening abnormalities across the vascular, renal, and peripheral nerve domains remain less well characterized. Importantly, a single current HbA1c measurement does not capture the long-term glycemic trajectory, and treatment intensification may lower current HbA1c after organ abnormalities have already developed. Individuals with similar current HbA1c values may therefore have substantially different prior glycemic exposure and existing organ burden (12). Accordingly, current HbA1c alone may not adequately identify patients with concurrent abnormalities in multiple organ domains, whereas readily available clinical information, including age, diabetes duration, obesity, and blood pressure, may provide additional risk information (8, 13). Determining whether these routinely available characteristics improve identification of this clustering phenotype beyond HbA1c alone may help clarify the role of current glycemic status within established screening practice.

We used the National Metabolic Management Center (MMC) registry to describe the burden and combination patterns of vascular, renal, and peripheral nerve screening abnormalities in adults with T2DM. We then examined the cross-sectional associations of current HbA1c and routine clinical characteristics with the multiorgan phenotype and compared models containing different combinations of clinical information.

## 2 Methods

### 2.1 Study design and setting

We performed a single-center cross-sectional analysis of the prospectively maintained MMC clinical registry at Wenzhou Hospital of Integrated Traditional Chinese and Western Medicine, China. Demographic, clinical, laboratory, vascular, and peripheral nerve data were collected during standardized MMC visits. Assessments completed between January 10, 2022, and April 30, 2026, were eligible. For each patient, the index assessment was defined as the single standardized visit selected for analysis; all exposures, covariates, and organ-domain measurements used in this study were taken from that visit. The analysis was therefore cross-sectional. Prospective maintenance refers to how the registry data were collected, not to longitudinal follow-up of the study outcome.

The study was approved by the Ethics Committee of Wenzhou Hospital of Integrated Traditional Chinese and Western Medicine (approval no. 2022-K024) and was conducted in accordance with the Declaration of Helsinki. At registry enrollment, all patients provided written informed consent for participation in the registry study and use of their clinical data.

### 2.2 Study population

The inclusion criteria were age 18–80 years; T2DM diagnosed according to guideline-defined criteria (14); and enrollment in the MMC clinical registry with completion of a standardized assessment during the study period. The age range of 18–80 years reflected the prespecified eligibility criteria of the source MMC registry and its standardized assessment pathway rather than an exclusion introduced during the present analysis. Adults older than 80 years were therefore not represented in the source population. This age restriction is clinically relevant because adults in this age group may have a greater burden of diabetes-related complications, together with age-related arterial stiffening, declining renal function, peripheral nerve dysfunction, frailty, and multimorbidity. Consequently, the prevalence and interpretation of organ-domain screening abnormalities in adults older than 80 years may differ from those in the population included in this study.

The exclusion criteria were type 1 diabetes, gestational diabetes, other specific types of diabetes, current pregnancy, or uncertain diabetes classification.

If a patient had undergone multiple assessments, one index assessment was selected according to a prespecified hierarchical rule: priority was given first to the record with complete outcome ascertainment and then to the record with more complete covariate data.

### 2.3 Clinical measurements and definitions of organ-domain abnormalities

#### 2.3.1 General characteristics

Age and sex were obtained from the standardized registry record at the index assessment. Diabetes duration was the number of years from the clinically documented diagnosis of T2DM to the index assessment. Weight, height and systolic blood pressure were the values recorded as part of the same standardized assessment. Venous blood collected at the index assessment was used to measure HbA1c by high-performance liquid chromatography using a Tosoh G8 analyzer (Tosoh Corporation, Japan) according to the manufacturer’s instructions. The value measured at the index assessment was defined as current HbA1c. BMI was calculated as weight in kilograms divided by height in meters squared. Diabetes duration, BMI, systolic blood pressure, and current HbA1c were analyzed as continuous variables in the primary regression model rather than dichotomized using abnormality cutoffs.

#### 2.3.2 Vascular domain

The vascular domain was assessed using brachial-ankle pulse wave velocity (baPWV) in combination with the ankle-brachial index (ABI). After at least 15 minutes of supine rest in a quiet, temperature-controlled room, baPWV and ABI were measured by trained staff using an automated waveform analyzer (BP-203RPE III, Omron Healthcare, Kyoto, Japan). Cuffs were placed on both upper arms and ankles, and bilateral brachial and ankle pressure waveforms were recorded simultaneously. Right and left baPWV and ABI values were calculated automatically by the device. Mean bilateral baPWV was calculated as the arithmetic mean of the valid right- and left-sided measurements. A vascular screening abnormality was defined as a mean bilateral baPWV ≥1400 cm/s, an ABI ≤0.90 in either limb, or an ABI >1.40 in either limb. baPWV has good reproducibility and has been used to assess arterial stiffness and cardiovascular risk (15–17); early research proposed approximately 1400 cm/s as an operational threshold for risk screening (18). However, baPWV is strongly influenced by age, blood pressure, and population composition. Other studies have reported risk thresholds above 1400 cm/s and age- and sex-specific reference ranges (19, 20). We therefore treated 1400 cm/s as a prespecified operational screening threshold rather than a diagnostic cutoff applicable across all ages and blood pressure levels. ABI thresholds were defined in accordance with contemporary guidelines for lower-extremity peripheral artery disease (21).

#### 2.3.3 Renal domain

The urinary albumin-to-creatinine ratio (UACR) was calculated from urinary albumin and urinary creatinine in a first-morning urine specimen collected at the index assessment. Urinary albumin was measured by immunoturbidimetry and urinary creatinine by enzymatic method; UACR was expressed in mg/g. Serum creatinine was measured by enzymatic method, and the estimated glomerular filtration rate (eGFR) was calculated using the 2021 race-free Chronic Kidney Disease Epidemiology Collaboration (CKD-EPI) creatinine equation (22). A renal screening abnormality was defined as a urinary albumin-to-creatinine ratio ≥30 mg/g or an eGFR <60 mL/min/1.73 m² (2, 23). Because chronic persistence cannot be established from a single assessment, we use the term renal screening abnormality rather than chronic kidney disease.

#### 2.3.4 Peripheral nerve domain

Peripheral nerve screening data were obtained from the standardized peripheral neuropathy examination report completed during the index assessment. The report provided an integrated classification based on measurements obtained with the SUDOSCAN-C1 electrochemical skin conductance analyzer. This method is simple, noninvasive, and rapid. We abstracted the final classification from the source report: patients classified as “abnormal” were considered to have a peripheral nerve screening abnormality, whereas those classified as “normal” were considered not to have such an abnormality. Reports that did not permit an unambiguous normal or abnormal classification were excluded from the analysis.

### 2.4 Outcome measures and candidate clinical variables

The primary outcome was prespecified as screening abnormalities in at least two of the three organ domains. We chose this definition to represent coexisting abnormalities beyond a single isolated finding; one abnormal domain alone was not considered multiorgan involvement. Secondary descriptive outcomes were the status of each organ domain, the number of abnormal domains (0–3), and eight mutually exclusive combinations of organ abnormalities. Current HbA1c, analyzed continuously, was the primary exposure. HbA1c <7.0% was the prespecified principal clinical threshold (24) and identified patients below the general treatment target commonly used for most nonpregnant adults. Candidate clinical variables were restricted to routinely available characteristics that were not part of the organ-domain definitions: current HbA1c, age, sex, diabetes duration, BMI, and systolic blood pressure. The measurements defining vascular, renal, and peripheral nerve abnormalities were not entered into the models, because doing so would reuse components of the outcome as predictors.

### 2.5 Statistical analysis

Continuous variables are presented as mean (standard deviation) or median [interquartile range], and categorical variables as number (percentage). Between-group comparisons according to the presence or absence of multiorgan abnormalities were performed using Welch’s t test, the Mann–Whitney U test, or the chi-square test, as appropriate. Vascular, renal, and peripheral nerve abnormalities were components of the primary outcome and were therefore reported descriptively only. Wilson score 95% confidence intervals (95% CIs) were calculated for binary outcomes and the proportions of individual organ abnormalities. Patients were grouped by HbA1c <7.0% versus ≥7.0% and were further stratified exploratorily into <7.0%, 7.0% to <8.0%, 8.0% to <9.0%, and ≥9.0% solely to describe the distribution of multiorgan abnormalities. A multivariable logistic regression model with multiorgan abnormalities as the dependent variable simultaneously included HbA1c, age, sex, diabetes duration, BMI, and systolic blood pressure. Continuous variables were modeled continuously, and ORs were reported per 1-percentage-point increase in HbA1c, 10-year increase in age, 5-year increase in diabetes duration, 5-kg/m² increase in BMI, and 10-mmHg increase in systolic blood pressure. Three nested models were prespecified: Model 1 included HbA1c alone; Model 2 additionally included age, sex, and diabetes duration; and Model 3 further included BMI and systolic blood pressure. Internal validation used 20 repeats of stratified fivefold cross-validation. Repeated out-of-fold predicted probabilities were averaged for each patient and used to calculate the AUC, Brier score, calibration intercept, and calibration slope. Patient-level out-of-fold predictions were then used in 2,000 paired bootstrap resamples to estimate 95% confidence intervals for the AUCs and between-model differences in AUC. To assess the potential impact of incomplete outcome ascertainment and associated selection bias, available baseline characteristics were compared between the 674 participants included in the primary analysis and the 57 excluded participants using variable-appropriate tests and absolute standardized mean differences (SMDs). An absolute SMD <0.10 was considered to indicate a small observed difference. As a sensitivity analysis, multiple imputation by chained equations was performed in the full eligible cohort of 731 participants, generating 50 imputed datasets. Missing current HbA1c was imputed using predictive mean matching. Missing binary organ-domain indicators were imputed using logistic regression, as applicable. The prespecified composite outcome of abnormalities in at least two organ domains was re-derived within each imputed dataset rather than imputed directly. Prevalence estimates and multivariable regression coefficients were pooled across imputations using Rubin’s rules. The three prespecified identification models were also re-evaluated within each imputed dataset using the same repeated stratified fivefold cross-validation procedure. AUCs and between-model differences in AUC were pooled across imputations using Rubin’s rules, incorporating within-imputation paired-bootstrap variance. All tests were two-sided, and P < 0.05 was considered statistically significant. Statistical analyses were performed using R version 4.5.3 and Python version 3.12. Reporting followed the STROBE and TRIPOD+AI guidelines (25, 26).

## 3 Results

### 3.1 Participant selection and characteristics at the index assessment

Of 731 patients with T2DM assessed for eligibility, 57 were excluded because current HbA1c was missing and/or data from at least one organ domain were insufficient to determine the primary outcome. Available characteristics were broadly comparable between included and excluded participants: absolute SMDs ranged from 0.005 to 0.079, and no between-group comparison was statistically significant (**Supplementary Table S1**). The remaining 674 patients were included in the analysis (**Figure 1**). There were 424 men (62.9%), the mean age was 52.6 ± 12.5 years, and the median observed diabetes duration was 6.0 [2.0–10.4] years. Patients with abnormalities in at least two organ domains were older and had longer diabetes duration, higher systolic and diastolic blood pressure, and lower BMI than those with fewer than two abnormal domains. Mean current HbA1c was similar between the groups (9.4% vs 9.1%; *P = 0.181*). **Table 1** gives the full demographic, clinical, and organ-screening characteristics.

**Figure 1:**
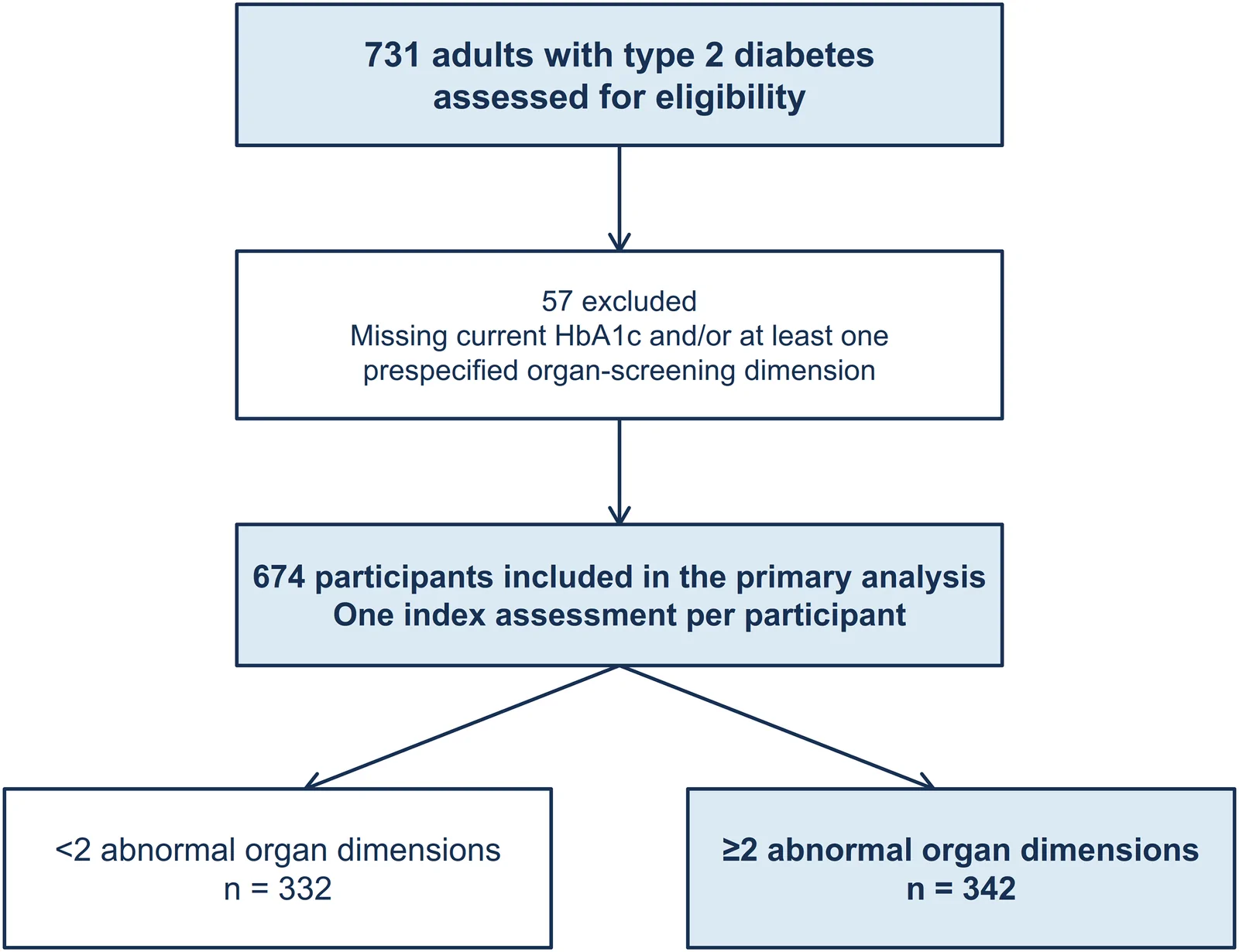
Participant selection flowchart. A total of 731 patients with T2DM were assessed for eligibility. After excluding 57 patients with missing current HbA1c and/or insufficient data for at least one prespecified organ domain, 674 patients were included in the primary analysis. HbA1c, glycated hemoglobin A1c.

**Table 1:** Demographic, Clinical, and Organ-Screening Characteristics at the Index Assessment.

Characteristic	Overall (n = 674)	<2 abnormal domains (n = 332)	≥2 abnormal domains (n = 342)	P value
Age, years	52.6 (12.5)	47.9 (11.6)	57.1 (11.6)	<0.001
Male sex, n (%)	424 (62.9)	226 (68.1)	198 (57.9)	0.006
Diabetes duration, years	6.0 [2.0–10.4]	4.0 [1.0–8.0]	9.0 [3.0–15.0]	<0.001
BMI, kg/m²	25.8 (4.1)	26.3 (4.4)	25.4 (3.8)	0.004
Systolic blood pressure, mmHg	133.3 (18.8)	127.3 (16.6)	139.0 (19.0)	<0.001
Diastolic blood pressure, mmHg	82.2 (11.0)	80.0 (10.1)	84.3 (11.5)	<0.001
Current HbA1c, %	9.3 (2.3)	9.1 (2.4)	9.4 (2.2)	0.181
Current HbA1c <7.0%, n (%)	112 (16.6)	68 (20.5)	44 (12.9)	0.008
Mean baPWV, cm/s	1533.5 [1360.2–1781.4]	1377.8 [1261.4–1556.0]	1677.2 [1508.0–1947.0]	<0.001
ABI ≤0.90 or >1.40, n (%)	18 (2.7)	4 (1.2)	14 (4.1)	0.020
UACR, mg/g	18.3 [8.9–47.4]	11.1 [6.8–18.7]	40.8 [17.0–131.3]	<0.001
SCr, μmol/L	63.5 [53.0–74.0]	63.0 [54.0–72.0]	64.0 [52.0–76.0]	0.292
eGFR, mL/min/1.73 m²	105.7 [98.2–114.6]	110.0 [103.0–118.5]	102.2 [94.3–110.5]	<0.001
Vascular screening abnormality^*^, n (%)	472 (70.0)	146 (44.0)	326 (95.3)	—
Renal screening abnormality^*^, n (%)	240 (35.6)	21 (6.3)	219 (64.0)	—
Peripheral nerve screening abnormality^*^, n (%)	324 (48.1)	64 (19.3)	260 (76.0)	—

Data are presented as mean (standard deviation), median [interquartile range], or n (%). P values were calculated using Welch’s t test, the Mann–Whitney U test, or the chi-square test, as appropriate for the variable type. *Vascular, renal, and peripheral nerve abnormalities were components of the primary outcome and are reported descriptively only; P values were not calculated.

ABI, ankle-brachial index; baPWV, brachial-ankle pulse wave velocity; BMI, body mass index; eGFR, estimated glomerular filtration rate; HbA1c, glycated hemoglobin A1c; SCr, serum creatinine; UACR, urinary albumin-to-creatinine ratio.

### 3.2 Overall burden and combination patterns of concurrent multiorgan screening abnormalities

Among the 674 patients in the analysis cohort, vascular, renal, and peripheral nerve screening abnormalities were present in 472 (70.0%), 240 (35.6%), and 324 (48.1%) patients, respectively. Overall, 342 patients (50.7%; 95% CI, 47.0%–54.5%) had abnormalities in at least two organ domains; 221 (32.8%) had abnormalities in two domains, and 121 (18.0%) had abnormalities in all three domains (**Figure 2A**). Among the specific combinations, the most common single-organ phenotype was an isolated vascular abnormality (146 patients, 21.7%). The most common multiorgan phenotype was combined vascular and peripheral nerve abnormalities (123 patients, 18.2%), followed by abnormalities in all three organ domains (121 patients, 18.0%) (**Figure 2B**).

**Figure 2:**
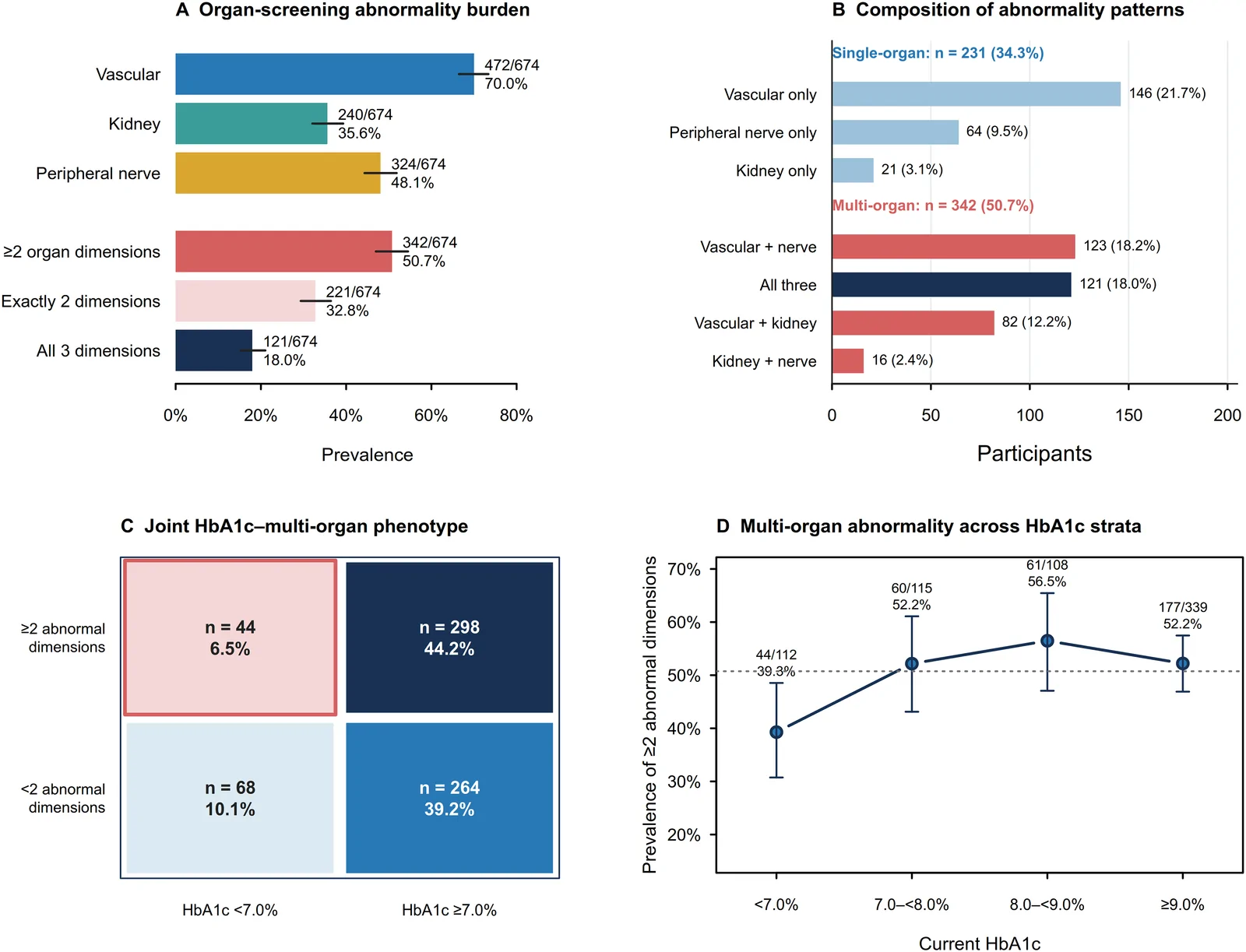
Burden and combination patterns of concurrent organ-domain abnormalities. **(A)** Prevalence of vascular, renal, peripheral nerve, and multiorgan abnormalities; error bars represent Wilson 95% CIs. **(B)** Composition of mutually exclusive organ-abnormality patterns. **(C)** Joint phenotype plot of HbA1c and multiorgan abnormalities using 7.0% as the principal clinical threshold. **(D)** Proportion of patients with abnormalities in at least two organ domains across HbA1c categories of <7.0%, 7.0% to <8.0%, 8.0% to <9.0%, and ≥9.0%; numbers above the bars indicate events/sample size. Unless otherwise stated, percentages were calculated using the entire analysis population (N = 674) as the denominator. HbA1c, glycated hemoglobin A1c.

### 3.3 Phenotypic distribution of multiorgan abnormalities across current HbA1c levels

When patients were grouped according to the primary clinical HbA1c threshold of 7.0%, 44 of the 112 patients with HbA1c <7.0% had abnormalities in at least two organ domains (39.3%, 95% CI 30.7%–48.5%), whereas 68 (60.7%) did not. Among the 562 patients with HbA1c ≥7.0%, 298 (53.0%, 95% CI 48.9%–57.1%) had multiorgan abnormalities, while 264 patients (39.2%) had HbA1c ≥7.0% but did not have multiorgan abnormalities (**Figure 2C**).

To further characterize the phenotypic distribution of multiorgan abnormalities at HbA1c levels ≥7.0%, this group was subdivided into 7.0% to <8.0%, 8.0% to <9.0%, and ≥9.0%. These three groups included 115, 108, and 339 patients, respectively, of whom 60, 61, and 177 had multiorgan abnormalities. The corresponding proportions were 52.2% (95% CI, 43.1%–61.1%), 56.5% (95% CI, 47.1%–65.4%), and 52.2% (95% CI, 46.9%–57.5%). When considered together with the HbA1c <7.0% group, the proportion with multiorgan abnormalities did not increase monotonically across the four current HbA1c categories in the descriptive analysis (**Figure 2D**).

### 3.4 Multivariable cross-sectional associations of current HbA1c and routine clinical characteristics with multiorgan abnormalities

When current HbA1c was entered as a continuous variable in the prespecified multivariable model, each 1-percentage-point increase showed no clear independent association with concurrent multiorgan abnormalities (adjusted OR, 1.04; 95% CI, 0.96–1.13; P = 0.296). In the same model, the adjusted ORs were 1.59 (95% CI, 1.34–1.89) per 10-year increase in age, 1.43 (95% CI, 1.20–1.71) per 5-year increase in diabetes duration, and 1.45 (95% CI, 1.30–1.62) per 10-mmHg increase in systolic blood pressure. Sex and BMI showed no clear adjusted cross-sectional associations with the outcome (**Figure 3**).

**Figure 3:**
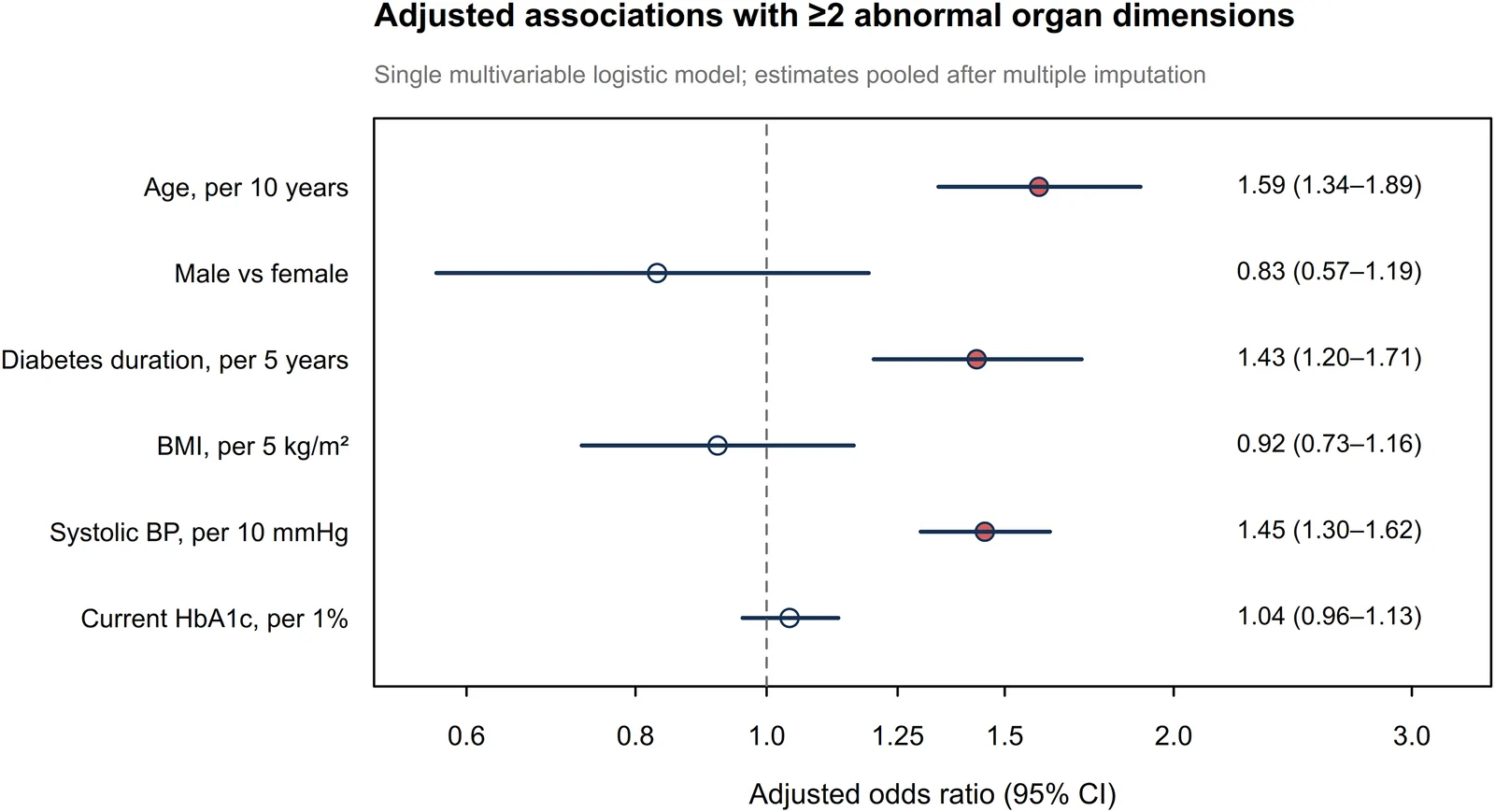
Adjusted associations with concurrent abnormalities in at least two organ domains. ORs were obtained from a single multivariable logistic regression model that simultaneously included all six prespecified variables. BMI, body mass index; BP, blood pressure; CI, confidence interval; HbA1c, glycated hemoglobin A1c; OR, odds ratio.

### 3.5 Incremental identification value of routine clinical characteristics beyond current HbA1c

When current HbA1c alone was used to identify concurrent multiorgan abnormalities, discrimination was close to chance (cross-validated AUC, 0.523; 95% CI, 0.479–0.568; Brier score, 0.250). After the addition of age, sex, and diabetes duration, the AUC increased to 0.732 (95% CI, 0.694–0.768), and the Brier score decreased to 0.209. The full model, which further included BMI and systolic blood pressure, achieved an AUC of 0.775 (95% CI, 0.740–0.810), representing an absolute improvement of 0.251 over the HbA1c-only model (95% CI, 0.195–0.304; *P = 0.001*). The full model had a Brier score of 0.194, a calibration intercept of −0.003, and a calibration slope of 0.943 (**Table 2**; **Figure 4**). In the multiple-imputation sensitivity analysis including all 731 eligible participants, results were consistent with the primary analysis. The pooled estimated prevalence of abnormalities in at least two organ domains was 50.7% (95% CI, 47.0%–54.4%), compared with 50.7% in the primary analysis. The adjusted association of current HbA1c with the outcome remained weak (OR per 1-percentage-point increase, 1.05; 95% CI, 0.97–1.14), whereas age (OR per 10 years, 1.57; 95% CI, 1.32–1.87), diabetes duration (OR per 5 years, 1.50; 95% CI, 1.25–1.79), and systolic blood pressure (OR per 10 mmHg, 1.47; 95% CI, 1.31–1.64) remained positively associated with the outcome. Internal-validation results were also similar: pooled AUCs were 0.538 (95% CI, 0.494–0.581), 0.740 (95% CI, 0.703–0.778), and 0.785 (95% CI, 0.751–0.820) for Models 1, 2, and 3, respectively, with a Model 3 versus Model 1 difference in AUC of 0.248 (95% CI, 0.196–0.300) (**Supplementary Table S2**).

**Table 2:** Internal Validation Performance of the Prespecified Identification Models.

Model	Predictors	AUC (95% CI)	Difference in AUC vs model 1	Brier score	Calibration intercept	Calibration slope
Model 1	Current HbA1c	0.523 (0.479–0.568)	Reference	0.250	0.019	0.363
Model 2	Model 1 + age + sex + diabetes duration	0.732 (0.694–0.768)	0.209 (0.154–0.263)	0.209	−0.001	0.949
Model 3	Model 2 + BMI + systolic blood pressure	0.775 (0.740–0.810)	0.251 (0.195–0.304)	0.194	−0.003	0.943

Model performance was estimated from the mean out-of-fold predictions obtained using 20 repeats of stratified fivefold cross-validation. The 95% CIs and differences in AUC were estimated using patient-level bootstrap resampling with 2,000 iterations.

AUC, area under the receiver operating characteristic curve; BMI, body mass index; CI, confidence interval; HbA1c, glycated hemoglobin A1c.

**Figure 4:**
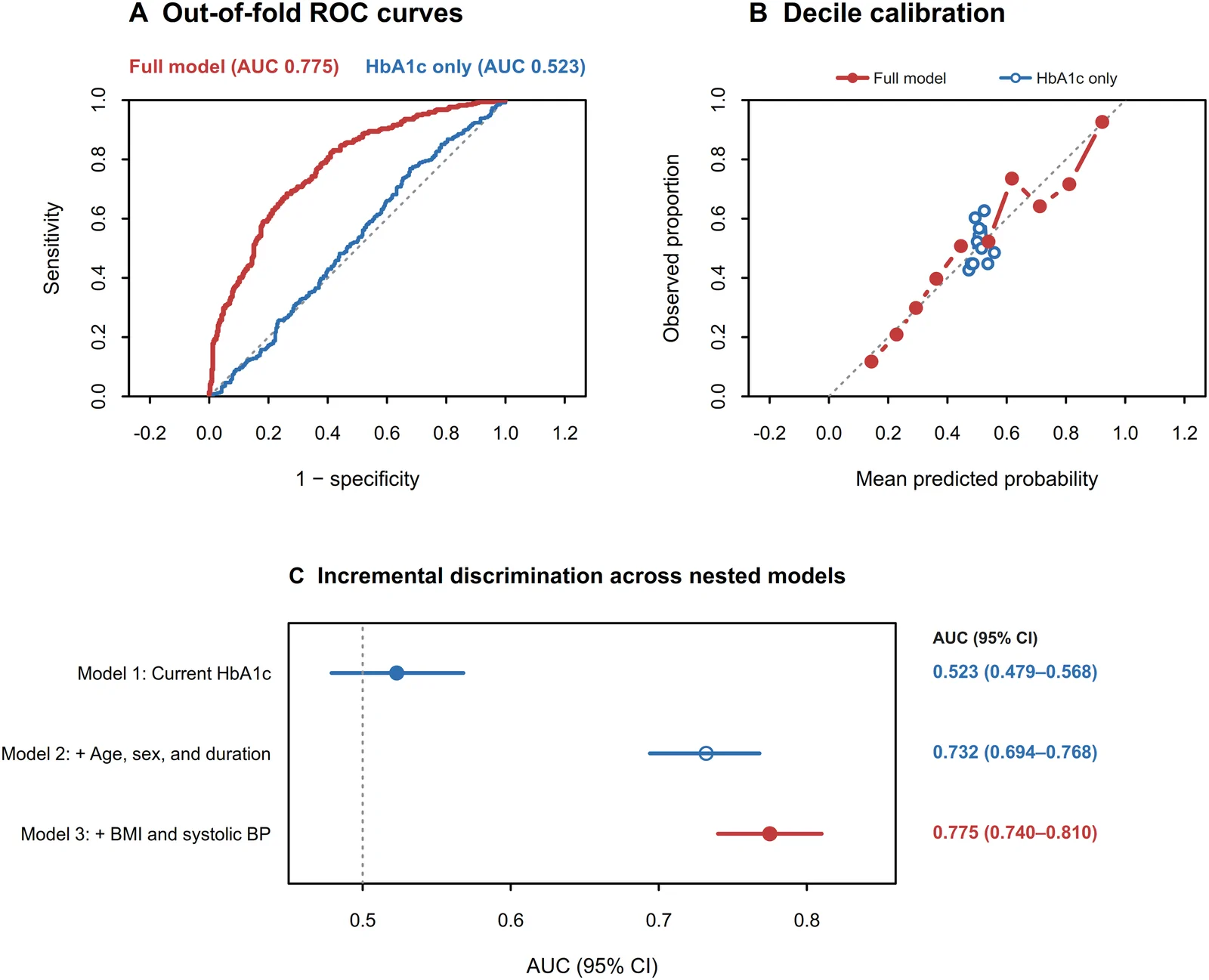
Incremental identification beyond current HbA1c. **(A)** Receiver operating characteristic curves based on out-of-fold predictions. **(B)** Calibration plot by deciles of predicted probability. **(C)** AUCs of the nested models. AUC, area under the receiver operating characteristic curve; BMI, body mass index; CI, confidence interval; HbA1c, glycated hemoglobin A1c; ROC, receiver operating characteristic.

## 4 Discussion

In this cross-sectional analysis of the MMC registry, 50.7% of patients with T2DM had screening abnormalities in at least two organ domains. Combined vascular and peripheral nerve abnormalities and involvement of all three domains were the most frequent patterns. The adjusted association between current HbA1c and the multiorgan phenotype was weak, and the proportion affected did not rise monotonically across HbA1c categories. Even among patients with HbA1c <7.0%, 39.3% had multiorgan abnormalities. Age, diabetes duration, and systolic blood pressure, by contrast, were independently associated with the outcome. Adding these variables, sex, and BMI to HbA1c increased the AUC from 0.523 to 0.775 (ΔAUC = 0.251). Thus, routinely collected clinical information helped distinguish patients with concurrent multiorgan abnormalities, whereas a single HbA1c value did not reflect the full burden of established organ abnormalities.

The weak association observed between current HbA1c and clustering of multiorgan abnormalities does not contradict the importance of long-term glycemic control in preventing complications. Multiple previous studies have demonstrated sustained benefits from maintaining good long-term HbA1c control (10, 11, 24). Several factors may explain our findings. First, current HbA1c primarily reflects recent glycemia, whereas arterial stiffness, albuminuria, reduced glomerular filtration, and peripheral nerve abnormalities may result from cumulative metabolic exposure over a much longer period. Moreover, a substantial proportion of patients may already have microvascular or macrovascular complications when T2DM is diagnosed (27). Second, glycemic variability and within-day excursions may contribute to organ injury independently of mean HbA1c. Even among patients with well-controlled HbA1c, greater glycemic variability may be associated with microvascular and macrovascular injury through mechanisms involving oxidative stress, endothelial dysfunction, and inflammatory pathways (28, 29). In addition, treatment intensification after detection of an organ abnormality may lower subsequent current HbA1c, giving rise to confounding by indication or reverse causation. Blood pressure burden, dyslipidemia, BMI, and lifestyle factors may also independently contribute to organ burden (30). Together, these factors limit the ability of a single HbA1c measurement to capture established abnormalities across multiple organ systems.

Diabetes-related organ abnormalities can coexist, but their frequencies are often discordant (6, 7). A large study from China, for example, found that diabetic kidney disease and retinopathy did not always occur together (8). This is consistent with our findings, suggesting that a normal screening result in one organ domain does not guarantee that other domains are unaffected. The clustering of multiorgan abnormalities may capture the overall burden of existing disease in patients with T2DM more comprehensively than any single-organ measure. The definition used here—abnormalities in at least two organ domains—is an operational phenotype for describing coexisting abnormalities, not a diagnosis of a distinct diabetic complication.

Age, diabetes duration, and systolic blood pressure added information beyond current HbA1c. This is clinically plausible: age and diabetes duration partly represent the time over which risk has accumulated, whereas systolic blood pressure captures an important nonglycemic burden (30–32). Each 10-mmHg increase in systolic blood pressure was associated with 45% higher odds of the multiorgan phenotype, similar in scale to the estimates for diabetes duration (OR, 1.43 per 5 years) and age (OR, 1.59 per 10 years). Randomized trials have established blood pressure control as part of comprehensive risk-factor management in T2DM (33). baPWV has been associated with peripheral neuropathy in patients with T2DM (34, 35). Notably, baPWV is strongly influenced by age and blood pressure, and vascular-domain abnormalities were present in 70.0% of our cohort. Thus, part of the discriminatory value of age and systolic blood pressure may reflect their physiological coupling with the baPWV-based definition of vascular abnormality. Reported baPWV risk thresholds vary by population, age, and sex (18–20). Our results with a fixed cutoff of 1400 cm/s are therefore specific to this operational definition and should be tested in independent populations using other baPWV cutoffs or vascular definitions. BMI was not independently associated with multiorgan abnormalities in this study, possibly because of the effects of age, diabetes duration, treatment status, reverse causality, and residual confounding. Importantly, the absence of an independent association should not be interpreted as evidence that a lower BMI protects against multiorgan abnormalities in patients with T2DM.

Recent HbA1c and organ screening should be regarded as complementary, not interchangeable. Age, diabetes duration, and systolic blood pressure provide information that a current HbA1c value does not. Assessment of established organ abnormalities in T2DM should therefore take account of recent glycemia, disease duration, and other clinical risk factors. Our model has only been internally validated. It may help flag patients more likely to have abnormalities in several organ domains, but it cannot replace direct screening. Until external validation and clinical utility are established, it should not be used to decide who is or is not screened.

This study has several limitations. First, the cross-sectional design meant that HbA1c and all organ measures were obtained concurrently at a single visit, precluding determination of temporal sequence or causality. Current HbA1c reflects recent glycemia, whereas organ abnormalities may result from long-term cumulative metabolic exposure; established abnormalities may also prompt treatment intensification and a lower subsequent HbA1c. The observed association should therefore not be interpreted as causal or as evidence against the benefits of long-term glycemic control. Prospective studies incorporating serial HbA1c, historical glycemic trajectories, and continuous glucose monitoring are needed to establish temporal relationships. Second, the prespecified composite outcome combined abnormalities with heterogeneous mechanisms and measurement methods. Its clinical meaning should be evaluated in independent cohorts and alongside analyses of individual organ domains and specific combinations. Third, the vascular definition used a fixed baPWV threshold of 1400 cm/s, which is influenced by age and blood pressure; the present findings should not be generalized as a diagnostic standard for arterial stiffness and should be tested using age- and sex-specific thresholds or alternative vascular definitions. Fourth, the renal and peripheral nerve domains were each classified from a single screening assessment without longitudinal confirmation or a uniform electrophysiological endpoint, creating potential outcome misclassification. Future studies should use repeated renal measurements and standardized neurologic or electrophysiological assessments. Fifth, measures of long-term glycemic exposure, glucose-lowering and cardiorenal protective medications, smoking, lipid levels, and other potential confounders were unavailable, so residual confounding may have influenced the reported associations. Sixth, 57 of 731 patients (7.8%) were excluded because of incomplete HbA1c or organ-domain data. Although measured characteristics were broadly similar and sensitivity analyses supported the robustness of the regression coefficients and model performance, some residual selection bias may remain. Finally, the single-center setting, restriction to adults aged 18–80 years, and reliance on internal cross-validation limit generalizability to adults older than 80 years, other ethnic groups, and different levels of healthcare. Prospective multicenter external validation is required before clinical implementation.

## 5 Conclusions

In this single-center cross-sectional cohort, current HbA1c alone had limited ability to identify patients with concurrent abnormalities across the vascular, renal, and peripheral nerve screening domains. The finding that 39.3% of patients with HbA1c <7.0% had the multiorgan phenotype indicates that achievement of a current glycemic target should not be used to defer recommended renal and peripheral nerve screening or cardiovascular risk assessment. The improvement in discrimination after age, sex, diabetes duration, BMI, and systolic blood pressure were added supports further evaluation of a multivariable case-finding approach, but this internally validated model is not a prospective risk predictor and should not be used to determine screening eligibility or treatment. Multicenter prospective studies should evaluate transportability across populations, use repeated standardized organ measurements and longitudinal glycemic and treatment data, and determine whether model-informed assessment improves clinical decisions or patient outcomes.

## Data Availability

The original contributions presented in the study are included in the article/**Supplementary Material**. Further inquiries can be directed to the corresponding author.
