# Supplementary material for: Association of current HbA1c with concurrent multiorgan screening abnormalities and the incremental value of routine clinical characteristics in type 2 diabetes: a single-center cross-sectional study in Wenzhou, China
Source: Front Endocrinol (Lausanne). 2026 Oct 7;17:1974690. doi: 10.3389/fendo.2026.1974690 (PMC13683135; doi:10.3389/fendo.2026.1974690)
Supplement: Supplementary file 1 [file Table1.docx]

**Supplementary Material**

**Supplementary Table S1. Comparison of available characteristics between participants included in the primary analysis and those excluded because of incomplete data**

| **Characteristic** | **Included cohort (n = 674)** | **Excluded comparison sample (n = 57)** | **Absolute SMD** | **P value** |
| --- | --- | --- | --- | --- |
| Age, years | 52.57 (12.48) | 53.35 (14.23) | 0.058 | 0.689 |
| Male sex, n (%) | 424 (62.9%) | 36 (63.2%) | 0.005 | 0.970 |
| Diabetes duration, years | 6.00 [2.00, 11.06] | 6.00 [3.00, 10.00] | 0.058 | 0.976 |
| BMI, kg/m² | 25.80 (4.12) | 25.61 (3.55) | 0.050 | 0.701 |
| Systolic blood pressure, mmHg | 133.25 (18.76) | 134.11 (17.74) | 0.047 | 0.729 |
| Diastolic blood pressure, mmHg | 82.17 (11.03) | 81.75 (9.38) | 0.040 | 0.755 |
| Current HbA1c, % | 9.27 (2.29) | 9.08 (2.35) | 0.079 | 0.575 |
| Mean baPWV, cm/s | 1604.48 (352.11) | 1618.07 (382.35) | 0.037 | 0.840 |
| ABI ≤0.90 or >1.40, n (%) | 18 (2.7%) | 1 (2.9%) | 0.016 | 0.612 |
| UACR, mg/g | 18.30 [8.89, 47.37] | 17.13 [9.25, 43.25] | 0.027 | 0.815 |
| Serum creatinine, μmol/L | 66.44 (21.78) | 67.35 (18.35) | 0.045 | 0.724 |
| eGFR, mL/min/1.73 m² | 104.20 (17.98) | 103.34 (17.51) | 0.048 | 0.726 |

**Notes:** Data are mean (standard deviation), median [interquartile range], or n (%). P values were calculated using Welch's t test, the Mann-Whitney U test, the chi-square test, or Fisher's exact test, as appropriate.

Absolute standardized mean differences (SMDs) were calculated from means or proportions. An absolute SMD <0.10 is conventionally considered a small observed difference.

All absolute SMDs were <0.10, suggesting no substantial observed imbalance in the measured characteristics; however, residual selection bias related to unmeasured characteristics or unobserved organ status cannot be excluded.

**Abbreviations:** ABI, ankle-brachial index; baPWV, brachial-ankle pulse wave velocity; BMI, body mass index; eGFR, estimated glomerular filtration rate; HbA1c, glycated hemoglobin A1c; SMD, standardized mean difference; UACR, urinary albumin-to-creatinine ratio.

**Missing values** were 36 for Current HbA1c, 23 for Mean baPWV, 23 for ABI, 9 for UACR, 9 for Serum creatinine, and 9 for eGFR.

**Supplementary Table S2. Sensitivity analysis of internal validation performance after multiple imputation in the full cohort (N = 731)**

**Primary-outcome prevalence:** After multiple imputation, the pooled prevalence of abnormalities in at least two organ domains was 50.7% (95% CI, 47.0%–54.4%), compared with 342/674 (50.7%; 95% CI, 47.0%–54.5%) in the primary analysis.

| **Model** | **Predictors** | **AUC (95% CI)** | **Difference in AUC vs Model 1 (95% CI)** | **Brier score** | **Calibration intercept** | **Calibration slope** |
| --- | --- | --- | --- | --- | --- | --- |
| Model 1 | Current HbA1c | 0.538 (0.494–0.581) | Reference | 0.249 | 0.007 | 0.690 |
| Model 2 | Model 1 + age + sex + diabetes duration | 0.740 (0.703–0.778) | 0.203 (0.149–0.257) | 0.206 | −0.001 | 0.961 |
| Model 3 | Model 2 + BMI + systolic blood pressure | 0.785 (0.751–0.820) | 0.248 (0.196–0.300) | 0.189 | −0.002 | 0.952 |

**Notes:** Fifty imputed datasets were generated using multiple imputation by chained equations in the full eligible cohort (N = 731). Missing current HbA1c was imputed using predictive mean matching. Missing binary organ-domain indicators were imputed using logistic regression, as applicable. The prespecified composite outcome of abnormalities in at least two of the three organ domains was not imputed directly; instead, it was re-derived from the completed organ-domain indicators within each imputed dataset. Within each imputed dataset, the three prespecified models were evaluated using 20 repeats of stratified fivefold cross-validation. AUCs and between-model AUC differences were pooled across imputations using Rubin’s rules, incorporating within-imputation paired-bootstrap variance. Brier scores and calibration estimates are reported as averages across imputed datasets.

For comparison, the corresponding primary-analysis AUCs in the 674 participants with observed outcome status were 0.523, 0.732, and 0.775 for Models 1, 2, and 3, respectively.

**Abbreviations:** AUC, area under the receiver operating characteristic curve; BMI, body mass index; CI, confidence interval; HbA1c, glycated hemoglobin A1c.
